# Postprandial metabolic effects of milk and yoghurt in young and older adults

**DOI:** 10.1186/s12263-025-00780-x

**Published:** 2025-10-13

**Authors:** Elaine Hillesheim, Gaïa Lépine, Patrick Neuhaus, Kathryn J. Burton-Pimentel, Jinyoung Kim, Katherine Li, Ulrich Bütikofer, Charlotte Fleuti, Corinne Marmonier, Dominique Dardevet, Sergio Polakof, Guy Vergères

**Affiliations:** 1 Agroscope, CH-3003 Bern, Switzerland; 2 Unité de Nutrition Humaine, INRAE, Université Clermont Auvergne, Clermont-Ferrand, 63000 France; 3 CNIEL, 42 Rue de Châteaudun, Paris, 75009 France

**Keywords:** Fermented foods, Milk, Yoghurt, Ageing, Postprandial period, Dietary challenge, Glucose, Insulin, Triglycerides, Free fatty acids

## Abstract

**Background:**

Postprandial metabolism plays a key role in cardiometabolic health, and its impairment with ageing is associated with increased disease risk. While yoghurt consumption has been linked to improved fasting metabolic markers, its acute postprandial effects are less well understood, particularly in older adults. This study investigated whether yoghurt consumption influences age-related postprandial metabolic dysregulation.

**Methods:**

In a randomised crossover design, 14 young (20–35 years) and 14 older (65–80 years) healthy men consumed 600 mL of whole milk or yoghurt following an overnight fast. Biochemical markers (glucose, insulin, triglycerides, TNF-α, IL-6, GIP and ghrelin) were measured at baseline and up to six hours postprandially. Differences in lipid metabolism by age were further investigated by assessing free fatty acid (FFA) responses in the yoghurt phase only. Postprandial responses were analysed for time, age, product and interaction effects, and summarised using incremental area under the curve (iAUC) and incremental maximum concentration (iCmax).

**Results:**

Glucose and insulin responses were influenced by product (time × product, p < 0.05), with yoghurt resulting in significantly lower iCmax values compared with milk. In contrast, triglyceride responses were influenced by age (time × age, p < 0.05), with older adults exhibiting higher iAUC and iCmax, delayed peak concentrations and slower return to baseline levels, independently of the product consumed. No significant age × product or age × product × time interactions were observed for any biochemical marker. Among the 37 FFAs quantified in the yoghurt phase, seven – predominantly saturated and abundant in yoghurt – exhibited a significant time × age interaction, accompanied by higher iAUC or iCmax in older adults.

**Conclusions:**

Dairy fermentation improved postprandial glucose and insulin responses, whereas ageing predominantly affected lipid dynamics. Fermentation did not attenuate impairments in postprandial triglyceride metabolism in this acute setting. Profiling individual postprandial FFAs may enhance understanding of metabolic flexibility and inform personalised dietary strategies across the lifespan.

**Supplementary Information:**

The online version contains supplementary material available at 10.1186/s12263-025-00780-x.

## Background

Postprandial metabolism is fundamental for maintaining metabolic health, as it regulates glucose, lipid and inflammatory responses following food intake. The ability to manage these transient fluctuations efficiently reflects metabolic flexibility, which refers to the body's capacity to adjust fuel utilisation in response to nutrient availability [1]. Although fasting measurements of metabolic markers are commonly used for disease diagnosis, abnormal postprandial elevations in glucose and lipids are stronger predictors of cardiovascular events in both healthy and diseased populations [2–4]. Therefore, developing strategies that modulate these postprandial responses, while accounting for the factors that influence them, is essential for supporting long-term metabolic health. Among these factors, meal composition, food matrix, and age are particularly influential in shaping postprandial metabolic dynamics [5, 6].

Fermentation is a traditional food processing technique that alters the physicochemical and nutritional properties of foods. In dairy products, fermentation by lactic acid bacteria not only generates bioactive compounds but also modifies the food matrix and microbial composition, potentially affecting digestion kinetics and nutrient bioaccessibility [7, 8]. These combined modifications likely contribute to the metabolic benefits attributed to fermented dairy consumption, as frequently observed in both epidemiological and human intervention studies [9, 10]. Yoghurt, in particular, has been associated with improved glycaemic health, prompting the recent approval of a qualified health claim by the United States Food and Drug Administration, which states that consuming at least three servings per week may reduce the risk of type 2 diabetes, although that the supporting evidence remains limited [11]. While many studies have focused on the health effects of chronic yoghurt consumption, typically using fasting biochemical markers, its immediate postprandial metabolic effects remain underexplored. Investigating these acute responses may provide critical insights into the mechanisms through which fermented foods influence metabolic health.

Older adults often exhibit metabolic dysregulation due to a gradual decline in metabolic efficiency, characterised by prolonged postprandial hyperglycaemia, slower triglyceride clearance, elevated circulating free fatty acids (FFAs) and increased inflammatory markers, all of which contribute to a higher risk of cardiometabolic diseases [12–16]. These impairments worsen with age, as demonstrated by progressive increases in the postprandial responses of glucose, insulin and total FFAs across four age strata from 65 to 84 years following an oral glucose tolerance test [14]. Given that FFAs serve not only as substrates for energy metabolism but also as signalling molecules that influence insulin sensitivity, inflammation and lipid metabolism [17], their postprandial dynamics represent an important yet underexplored aspect of age-related metabolic dysregulation. Investigating how ageing alters FFA responses to food intake may provide novel insights into mechanisms underlying impaired metabolic flexibility. Dairy products are widely recommended in dietary guidelines to support balanced diets [18] and are particularly encouraged in older adults to help prevent osteoporosis and preserve muscle mass and strength [19, 20]. Exploring whether fermented dairy products can help attenuate age-related impairments in postprandial responses linked to cardiometabolic risk may inform new dietary strategies to enhance metabolic resilience with ageing.

We previously conducted a crossover randomised trial in young and older men to identify robust biomarkers of milk and yoghurt consumption [21]. Several metabolites, including lactose, galactitol and galactonate, differentiated the two products and emerged as potential biomarker candidates. However, other metabolites, such as specific FFAs, acylcarnitines and sphingosine-1-phosphate, did not retain their discriminative capacity in older participants. In the present study, we build on this trial to investigate postprandial markers of metabolic health, examining whether milk and yoghurt elicit distinct responses reflecting potential effects of fermentation and whether these responses are influenced by age. Specifically, we assessed postprandial responses related to glucose (glucose, insulin and glucose-dependent insulinotropic polypeptide [GIP], ghrelin) and lipid (triglycerides) homeostasis and inflammation (TNF-α and IL-6). In addition, we evaluated age-related differences in the postprandial kinetics of 37 individually quantified FFAs.

## Methods

### Study population

The study included 28 healthy men, allocated to two groups based on age: 14 young adults (YA) aged 20–35 years and 14 older adults (OA) aged 65–80 years. Potential participants were screened via telephone interview, followed by a medical assessment [22]. In brief, healthy, lactose-tolerant individuals with regular dairy consumption (2–4 portions/day), within the specified age range required, and with a BMI of 21–30 kg/m^2^ were recruited. Exclusion criteria included chronic or acute illness, a history of anaemia, lifestyle factors such as smoking > 5 cigarettes/day, alcohol consumption > 3 glasses/day, or engaging in > 6 h of sports activity per week; regular intake of nutritional supplements, blood donation within three months prior to enrolment, current participation in another clinical study, and inability to comply with the study protocol for any reason.

The study was approved by the Ethical Committee for Personal Protection Ile de France IV (protocol code: 2017-A02879-44) and registered at www.clinicaltrials.gov (NCT03500003). All participants provided written informed consent, and study procedures were conducted in accordance with the Declaration of Helsinki. Study visits took place at the Human Nutrition Research Centre of Auvergne (CRNH-A, Clermont-Ferrand, France) between July 2018 and March 2019.

### Study design

**Fig. 1:**
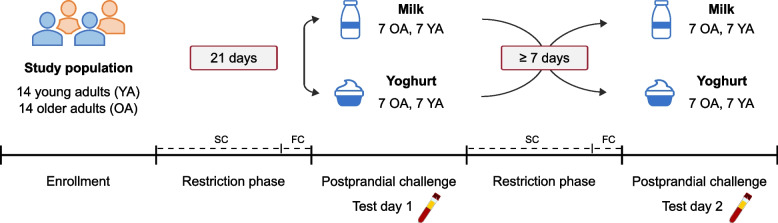
Study design. A randomised crossover trial investigating postprandial metabolic responses to full-fat milk and yoghurt challenges. During the restriction phases, participants followed semi-controlled and fully controlled diets in which they were instructed to exclude dairy products and limit fermented non-dairy foods. For each challenge, blood samples were collected at baseline (fasting state) and at nine postprandial time points (0.25, 0.5, 1, 1.5, 2, 3, 4, 5 and 6 h). FC, 2-day fully controlled diet; OA, older adults; SC, semi-controlled diet; YA, young adults

This manuscript presents secondary analyses of a randomised crossover trial investigating postprandial metabolic responses to full-fat milk and yoghurt challenges. The primary outcomes – serum and urine metabolomics to identify novel biomarkers of acute dairy intake – have been partially published [21, 22]. The study consisted of two phases: a restriction phase and a postprandial challenge phase (Fig. 1) [22]. During the 19-day restriction phase, participants followed a semi-controlled diet, excluding dairy products and limiting fermented non-dairy foods according to provided dietary guidance. In the two days preceding each postprandial challenge, participants were instructed to completely avoid dairy and fermented foods. On the first test day, following an overnight fast, participants arrived at the research centre and were randomly assigned to consume 600 mL of either full-fat milk or yoghurt, which was consumed within 15 min. The 600 mL dose was chosen based on the trial’s primary objective, aiming at maximising the postprandial effect of dairy intake and facilitating identification of associated metabolites. Randomisation was performed using an MS Excel function. Due to the nature of the intervention, neither participants nor investigators were blinded.

Blood samples were collected at baseline (fasting state) and at nine postprandial time points (0.25, 0.5, 1, 1.5, 2, 3, 4, 5 and 6 h). During the postprandial period, participants remained fasted. The procedure was repeated on a second test day with the alternate dairy product after a washout period of at least seven days, during which participants followed the semi-controlled diet for at least five days and the fully controlled diet in the two days preceding the second test day. For the present analyses investigating postprandial metabolic responses to milk and yoghurt challenges, only data from the two test days were used. Results from the restriction phase have been previously published [22].

### Challenge products

The challenge products were non-commercial, full-fat milk and yoghurt, manufactured in parallel from the same raw milk at the dairy research plant of Agroscope (Bern, Switzerland) [21]. In brief, the fat content of the raw milk was standardised to 3.6% through centrifugation. The standardised milk was then pasteurised and either heated to produce UHT milk or processed into yoghurt. For yoghurt production, milk was inoculated with the commercial mild starter culture YF-L 811 (Chr. Hansen, DK-2970 Horsholm), containing *Lactobacillus delbrueckii* subsp. *bulgaricus* and *Streptococcus thermophilus*, and fermented under controlled conditions. Both milk and yoghurt were stored in 150 g portions at 4 °C until the test days.

Samples from each product batch were collected prior to the test days and analysed for chemical and microbiological composition (ISO 7889 | IDF 117:2003) [23], with the results reported previously [21]. Both products had similar energy and protein contents (65 kcal and 3.3 g protein/100 g). Milk contained significantly more carbohydrates than yoghurt (4.8 vs 3.9 g/100 g, *p* < 0.001), whereas yoghurt had a slightly higher fat content (3.9 vs 3.7 g/100 g, p < 0.001). Yoghurt contained 2.3 × 10⁷ CFU/g of *S. thermophilus*, while *L. delbrueckii* subsp. *bulgaricus* was not detected in the range of 10^2^–10⁹ CFU/g. Microscopic analyses confirmed *S. thermophilus* as the dominant microorganism.

### Biochemical markers

Blood was collected from the antecubital vein using a 22G short catheter. Samples were immediately centrifuged (10 min, 3000 g, 4 °C), and the resulting serum was frozen in liquid nitrogen and stored at −80 °C until analysis. Glucose and triglyceride concentrations were measured using spectrophotometry on an automated chemistry analyser (ABX Pentra 400, Horiba, Montpellier, France). Enzyme-linked immunosorbent assay (ELISA) kits were used to measure insulin (Mercodia SAS, Paris, France), tumour necrosis factor-alpha (TNF-α; Invitrogen™ Human TNF-α ELISA Kit, Illkirch, France), interleukin-6 (IL-6; Human IL-6 Quantikine HS ELISA Kit, R&D Systems, Minneapolis, USA), glucose-dependent insulinotropic polypeptide (GIP; Human GIP (total) ELISA, Merck Millipore, St Quentin en Yvelines, France) and ghrelin (Invitrogen™ Human Ghrelin ELISA Kit, Illkirch, France). GIP and ghrelin measurements were performed without protease inhibitors; although this may have affected absolute concentrations, all samples were handled identically, ensuring that relative comparisons between groups remain valid. All markers were measured in the fasting state. Additionally, glucose, triglycerides and insulin concentrations were measured at nine postprandial time points (0.25, 0.5, 1, 1.5, 2, 3, 4, 5 and 6 h), while TNF-α, IL-6, GIP and ghrelin were measured at three postprandial time points (0.5, 1 and 3 h).

### Fatty acids

#### Serum 

Serum samples were collected at 0, 1, 2, 3, 4, 5 and 6 h, and analysed for FFAs in the yoghurt phase only. As triglyceride levels showed only age-related effects, with no differences between products, further investigation of age-related effects on lipid metabolism was carried out for yoghurt alone. FFAs were measured as previously described [22, 24], under conditions designed to minimise triglyceride hydrolysis [25, 26]. Briefly, 100 µL of serum was mixed with 30 µL of an internal standard solution (C13, 15 µg/30 µL), followed by methylation with 1 mL of 1.25 M methanol/HCl at 25 °C for 45 min. The reaction was neutralised by adding 350 µL of Na_2_CO_3_ solution (30% in water), and FFAs were extracted with 300 µL of hexane. The mixture was then vortexed and centrifuged at 1100 × g for 15 min. The organic supernatant was transferred to a vial, dried with 100 mg of Na_2_SO_4_, and vortexed and centrifuged again. A 0.5 µL aliquot of FFA solution was injected and analysed using high-resolution gas chromatography with a flame ionisation detector (GC-FID 6890 N, Agilent Technologies, Basel, Switzerland). A human serum control sample was analysed with each batch to monitor system performance, with C14 and C18:1 c9 concentrations plotted. Samples were analysed in five technical batches. In total, 67 individual FFAs were quantified, of which 37 were retained for analysis. FFAs were excluded if their concentrations were near the detection threshold, resulting in many missing values (*n* = 19), or if peak separation was insufficient for optimal quantification (*n* = 11).

#### Yoghurt

Ten samples from three yoghurt batches were analysed for individual fatty acids (FAs) in the total lipid fraction using a procedure adapted from ISO 15884:2002, which describes the preparation of fatty acid methyl esters from milk fat. For each sample, 0.3 g of total fat was weighed and mixed with 5 mL of an internal standard solution (C9). To maximise FA hydrolysis, mimicking gastrointestinal conditions, 0.25 mL of 2 mol/L KOH in methanol was added. The mixture was vortexed immediately until clear, and left to stand for 5 min. Subsequently, 0.5–0.6 g of NaHSO₄·H₂O was added to prevent saponification of the methyl esters. After vortexing, samples were centrifuged at 1100 × g for 15 min. A total of 3 µL of the organic supernatant was transferred to a vial and diluted with 1 mL of hexane. A 0.5 µL aliquot of FA solution was injected for GC-FID analysis, following the procedure described for serum samples [24]. A control sample was analysed with each batch to monitor system performance, with C4, C14 and C18:1 c9 concentrations plotted. The mean FA concentrations across the ten samples were taken as the representative value for yoghurt. In total, 67 FAs were quantified; however, as this study analysed FAs exclusively in relation to postprandial serum responses, only the 37 species retained in the serum analysis were considered for yoghurt.

### Statistical analysis

All analyses were performed using R 4.4.2 [27]. The normality of variables was assessed using the Shapiro–Wilk test and visual inspection of histograms and Q–Q plots. As most variables were not normally distributed, non-parametric tests were applied, and results are presented as medians with interquartile ranges. All data points, including statistical outliers, were included in the analyses. Postprandial responses were analysed as incremental concentrations relative to baseline to characterise responses independently of fasting status. The incremental area under the curve (iAUC) was calculated using the *auc* function (type = "linear") from the *MESS* package (version 0.5.12) [28], by summing both positive and negative incremental values across time points. The incremental maximum concentration (iCmax) was defined as the highest postprandial concentration observed for each individual, separately for each product challenge. The time at which this occurred was recorded as the time of maximum incremental concentration (iTmax). These indicators were analysed using the Wilcoxon signed-rank test for paired comparisons within age groups and between product challenges, and the Mann–Whitney U test for unpaired comparisons between age groups for the same product challenge.

Postprandial responses were analysed using Wald test statistics from the Nonparametric Analysis of Longitudinal Data in Factorial Experiments (*nparLD* package, version 2.2) [29]. Biochemical markers were evaluated using the *f1.ld.f2* function to assess the effects of age, product, and time, along with their two- and three-way interactions (age × product, time × product, age × time, and age × product × time). In this model, age and product were treated as whole-plot factors, and time as a sub-plot factor. As the model does not account for carryover effects, we assumed no carryover between test days (Supplementary Table 1). FFAs, quantified only in the yoghurt phase, were analysed using the *f1.ld.f1* function to assess the effects of age and time, and their interaction (age × time), with age as a whole-plot factor and time as a sub-plot factor.

Repeated measures correlations between postprandial incremental changes in triglycerides and FFA were performed using the *rmcorr* package (version 0.6.0) [29], which accounts for within-individual dependency. Six postprandial time points were considered and 95% confidence intervals for the correlations were generated using 1,000 non-parametric bootstrap simulations.

Statistical significance for biochemical marker analyses was set at *p* < 0.05, while p-values between 0.05 and 0.1 were considered trend effects. For FFA analyses, the Benjamini–Hochberg procedure was used to control for multiple testing within each effect type (nparLD analyses with 37 FFAs) and age groups (repeated measures correlation analyses with 7 FFAs), with significance set at FDR < 0.05. The *ComplexHeatmap* package (version 2.20.0) [30] was used to generate heatmaps, with rows clustered using Euclidean distance. All other plots were produced with the *ggplot2* package (version 3.5.1) [31] and final annotations were performed in Inkscape (version 1.0.2–2, Inkscape Project, New York, USA).

## Results

All 28 participants completed both milk and yoghurt challenges. Median ages were 27.5 years (IQR: 25.0–31.0) for YA and 69.0 years (IQR: 66.0–71.0) for OA. Although body weight was higher in YA compared with OA, BMI did not differ significantly due to the greater height in YA. Median baseline biochemical markers did not differ between age groups (Table 1) and between the baselines of each study visit (Supplementary Table 1), with median glucose, insulin and triglycerides concentrations within reference ranges for healthy individuals.

**Table 1:** Participants' characteristics at baseline by age group

	**Young adults** **(*n* = 14)**	**Older adults** **(*n* = 14)**	**P-value**
Age (years)	27.5 (25.0, 31.0)	69.0 (66.0, 71.0)	-
BMI (kg/m^2^)	25.1 (22.2, 25.9)	23.8 (22.5, 26.4)	0.910
Weight (kg)	78.4 (75.0, 83.3)	71.1 (66.9, 75.4)	0.056
Height (m)	1.79 (1.73, 1.85)	1.73 (1.69, 1.74)	0.009
Glucose (mmol/L)	4.83 (4.69, 5.16)	4.94 (4.75, 5.23)	0.597
Insulin (pmol/L)	25.3 (18.7, 30.4)	23.2 (15.5, 32.4)	0.571
Triglycerides (mmol/L)	0.73 (0.66, 0.86)	0.95 (0.80, 1.06)	0.124
TNF-α (pg/mL)	6.94 (5.92, 8.69)	5.73 (4.73, 7.91)	0.376
IL-6 (pg/mL)	0.84 (0.62, 1.34)	1.17 (0.89, 2.16) *	0.141
GIP (pg/L)	0.076 (0.048, 0.096)	0.065 (0.056, 0.092)	0.910
Ghrelin (pg/L)	3.91 (3.02, 5.86)	4.49 (2.14, 5.57)	0.874

Values are medians and interquartile ranges. Differences between age groups were assessed using the Mann–Whitney U test

*BMI* body mass index, *GIP* glucose-dependent insulinotropic polypeptide, *IL-6* interleukin-6, *TNF-α* tumour necrosis factor-alpha

*One missing value resulted in 13 measurements

### Yoghurt elicits lower glucose and insulin responses compared with milk

**Fig. 2:**
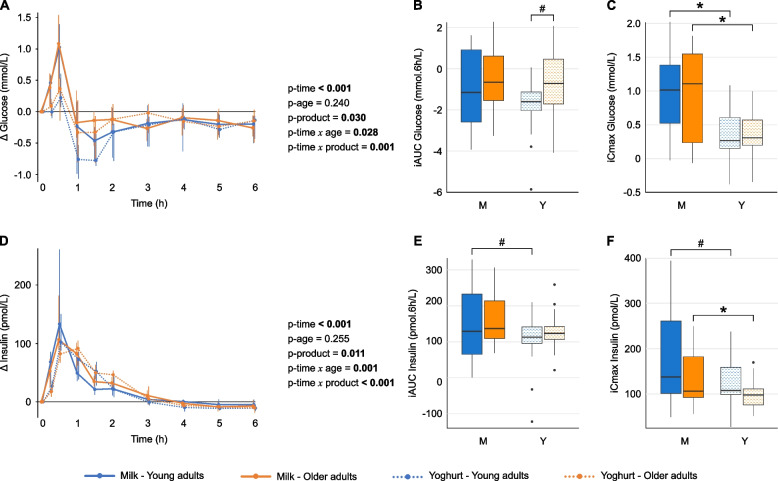
Postprandial glucose and insulin responses to milk and yoghurt challenges. Line plots (**A**, **D**) display median postprandial changes from baseline with IQRs. Intervention effects were assessed using the Wald test from the Nonparametric Analysis of Longitudinal Data in Factorial Experiments. All age × product and time × age × product interactions were non-significant (*p* > 0.05). Box-and-whisker plots display iAUC (**B**, **E**) and iCmax (**C**, **F**) with the median (horizontal line), IQR (the box), and the smallest and largest values within 1.5 times the IQR from the lower and upper quartiles (whiskers). Data points beyond this range are shown individually and represent statistical outliers. The Wilcoxon signed-rank test was used to compare responses between test products within age groups, while the Mann-Whitney U test was used to compare responses between age groups for the same test product. # *p* < 0.10, * *p* < 0.05. Line plots of absolute postprandial responses are provided in Supplementary Figure S6. iAUC, incremental area under the curve; iCmax, incremental maximum concentration; IQR, interquartile range; OA, older adults; YA, young adults

Analysis of postprandial glucose and insulin responses to milk and yoghurt challenges revealed significant effects of time, product and their interaction (time × product), indicating that responses differed between the products (Figs. 2A/D). Although differences in iAUC between milk and yoghurt were not statistically significant (Figs. 2B/E), yoghurt consumption resulted in a significantly lower glucose iCmax in both YA and OA, and a significantly lower insulin iCmax in OA, with a trend toward lower insulin iCmax in YA (Figs. 2C/F). For both markers, the most pronounced differences between product responses were observed within the first three hours following the dietary challenge (Supplementary Fig. 1). Despite a significant time × age interaction, indicating potential differences in temporal response between YA and OA, glucose and insulin peaked at 0.5 h post-challenge (iTmax) in both age groups, independent of the product (Supplementary Table 2).

### Triglyceride responses are higher in older adults compared with young adults, independently of product

**Fig. 3:**
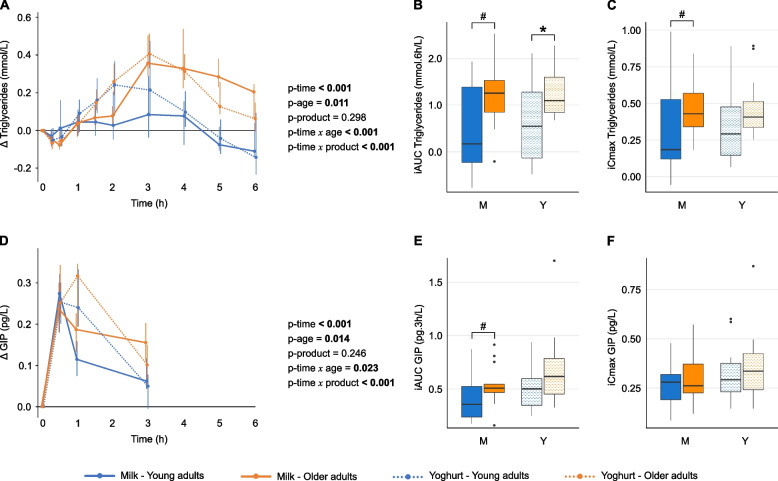
Postprandial triglycerides and GIP responses to milk and yoghurt challenges. Line plots (**A**, **D**) display median postprandial changes from baseline with IQRs. Intervention effects were assessed using the Wald test from the Nonparametric Analysis of Longitudinal Data in Factorial Experiments. All age × product and time × age × product interactions were non-significant (*p* > 0.05). Box-and-whisker plots display iAUC (**B**, **E**) and iCmax (**C**, **F**) with the median (horizontal line), IQR (the box), and the smallest and largest values within 1.5 times the IQR from the lower and upper quartiles (whiskers). Data points beyond this range are shown individually and represent statistical outliers. The Wilcoxon signed-rank test was used to compare responses between test products within age groups, while the Mann-Whitney U test was used to compare responses between age groups for the same test product. # *p* < 0.10, * *p* < 0.05. Line plots of absolute postprandial responses are provided in Supplementary Figure S6. GIP, glucose-dependent insulinotropic polypeptide; iAUC, incremental area under the curve; iCmax, incremental maximum concentration; IQR, interquartile range; OA, older adults; YA, young adults

Analysis of postprandial triglyceride responses to milk and yoghurt challenges revealed significant effects of time, age and their interaction (time × age), but no effect of product, indicating that responses differed between age groups irrespective of the dairy product consumed (Fig. 3A). Following the yoghurt challenge, OA exhibited significantly higher triglyceride iAUC than YA, with a trend towards higher values observed following the milk challenge (Fig. 3B). Likewise, a trend towards higher iCmax was observed in OA compared with YA following the milk challenge (Fig. 3C). For both products, iTmax was significantly delayed in OA compared with YA (Supplementary Table 2). As a result, the postprandial triglyceride response curves began to diverge between age groups from three hours onwards, with median incremental concentrations remaining consistently higher in OA than in YA up to six hours (Supplementary Fig. 2).

Similar to triglycerides, GIP exhibited significant time, age and time × age effects in postprandial analyses. Slightly higher iAUC values were observed in older adults, with a trend effect following the milk challenge. However, comparisons of iCmax between age groups did not reach statistical significance (Figs. 3D-F**,** Supplementary Table 2). Among the remaining biochemical markers, TNF-α showed a significant age effect without a corresponding time effect, whereas IL-6 and ghrelin exhibited significant time effects only, with no influence of age or product (Supplementary Fig. 3, Supplementary Table 2).

### FFA responses are also higher in older compared with young adults

**Fig. 4:**
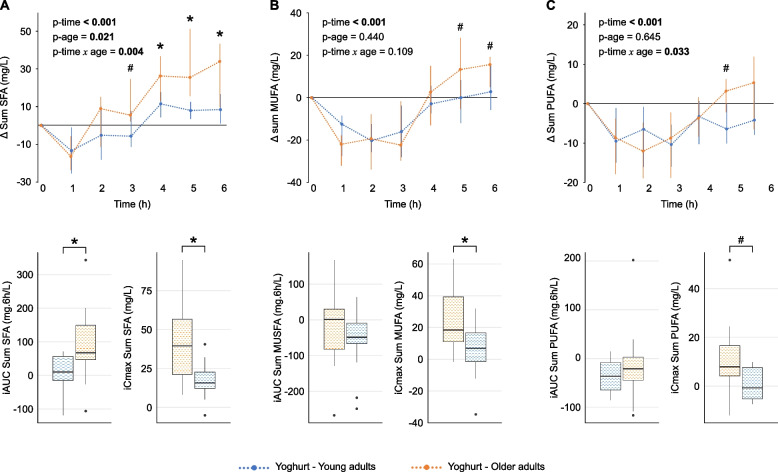
Postprandial responses of FFA classes to yoghurt challenge. Line plots (**A**, **B**, **C**) display median postprandial changes from baseline with IQRs. Intervention effects were assessed using the Wald test from the Nonparametric Analysis of Longitudinal Data in Factorial Experiments. Box-and-whisker plots display iAUC and iCmax with the median (horizontal line), IQR (the box), and the smallest and largest values within 1.5 times the IQR from the lower and upper quartiles (whiskers). Data points beyond this range are shown individually and represent statistical outliers. The Mann–Whitney U test was used to compare responses between age groups. # *p* < 0.10, * *p* < 0.05. Line plots of absolute postprandial responses are provided in Supplementary Figure S7. iAUC, incremental area under the curve; iCmax, incremental maximum concentration; IQR, interquartile range; MUFA, monounsaturated fatty acid; PUFA, polyunsaturated fatty acid; SFA, saturated fatty acid

Data on FFA responses were available for the yoghurt challenge only, allowing us to investigate the effect of age on postprandial responses. Of the 37 FFAs included in the analyses, 10 were saturated (SFA), 13 were monounsaturated (MUFA) and 14 were polyunsaturated (PUFA). At baseline, fasting concentrations of the sum of SFAs, MUFAs and PUFAs, as well as 5 individual FFAs, differed significantly between age groups (Supplementary Table 3). OA had higher concentrations of all sums and of C16:1 c9 (ω7), C18:1 c11 (ω7), C18:2 c9,c12 (ω6) and C18:3 c9,c12,c15 (ω3), but lower concentrations of C10 compared with YA. Analysis of postprandial responses revealed a significant time effect for the sums (Fig. 4) and for 32 individual FFAs (Supplementary Table 4), with median iAUCs being positive for the sum of SFAs and 20 individual FFAs and negative for the sum of PUFAs and 12 individual FFAs, in both age groups. While the median iAUC for the sum of MUFAs was clearly negative in YA, it was close to zero in OA. Concomitant age and time × age effects were observed for the sum of SFAs and for 7 individual FFAs, including 5 saturated (C10, C12, C14, C15 and C15 aiso) and 2 unsaturated (C14:1 c9 (ω5) and C18:1 t10 + t11). Significantly higher iAUCs were observed in OA for the sum of SFAs and 6 of these FFAs.

**Fig. 5:**
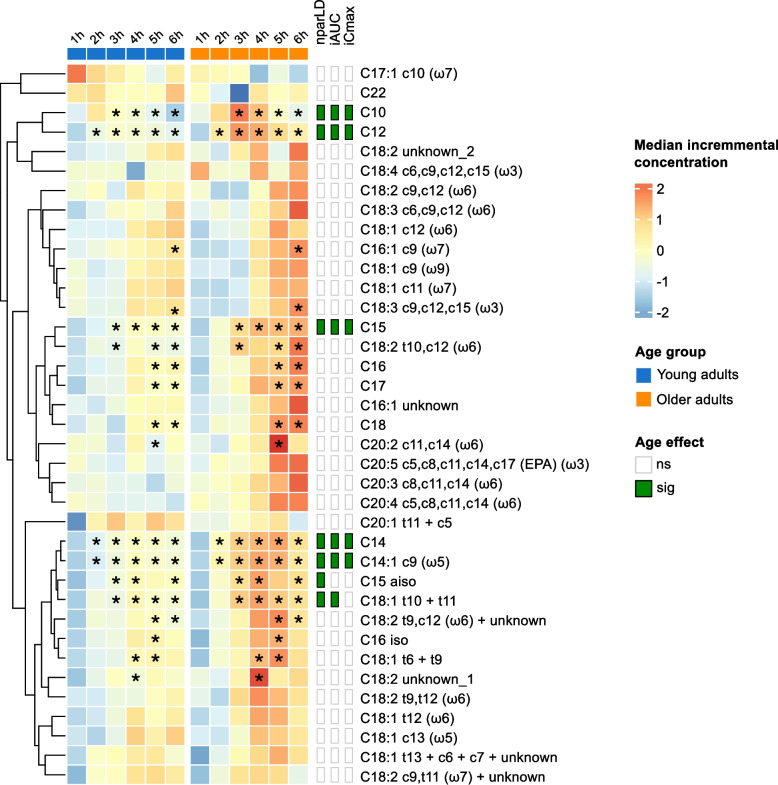
Postprandial responses of individual FFAs to yoghurt challenge. The heatmap displays median incremental concentrations of postprandial FFA responses, scaled by metabolite. Age effects (green annotation) denote significant differences between age groups as follows: nparLD indicates concomitant time, age and time × age effects; iAUC indicates a significant difference in iAUC; and iCmax indicates a significant difference in iCmax (FDR < 0.05). * Incremental median concentrations are significantly different between age groups for the specified FFA at the indicated time point (*p* < 0.05). Intervention effects were assessed using the Wald test from the Nonparametric Analysis of Longitudinal Data in Factorial Experiments (nparLD). The Mann–Whitney U test was used to compare median concentrations at each time point, iAUC and iCmax between age groups. FDR, false discovery rate; FFA, free fatty acid; iAUC, incremental area under the curve; iCmax, incremental maximum concentration

Analysis of postprandial response curves indicated that the two age groups began to diverge primarily from two hours onwards, with the sums (Fig. 4) and 18 individual FFAs (Fig. 5, Supplementary Fig. 4) showing significantly higher incremental concentrations at specific time points in OA compared with YA. Notably, 90% (*n* = 9) of the saturated FFAs analysed exhibited significant differences between age groups at one or more time points, whereas this was observed in 31% (*n* = 4) of monounsaturated and 36% (*n* = 5) of polyunsaturated FFAs. However, no differences in iTmax were observed between age groups for either the sums or individual FFAs.

Visual inspection of Fig. 5 revealed distinct clusters of FFA responses based on their postprandial trajectories, with some more pronounced among those showing age-related differences. Three main clusters were observed: (1) a pair of medium-chain saturated FFAs (C10 and C12) that exhibited rapid postprandial increases; (2) a mix of even- and odd-chain long-chain FFAs (C15, C16, C17, C18, C16:1 unknown, and C18:2 t10,c12 (ω6)) characterised by steadily rising and sustained elevations, particularly in older adults; and (3) a cluster enriched in branched-chain and trans-configured FFAs (C15 iso, C16 iso, C18:1 t6 + t9, and C18:2 t9,c12 (ω6) + unknown), together with C14 and C14:1 c9, which displayed more variable trajectories with delayed peaks.

Given the relationship between triglyceride and FFA metabolism, we investigated correlations between the incremental concentrations of triglycerides and the FFAs that showed a significant time × age effect (Supplementary Fig. 5). In OA, all the individual postprandial FFA responses were strongly correlated with triglyceride responses (rm = 0.382 to 0.828). In contrast, only C10 and C12 were significantly correlated with triglyceride responses in YA, with lower correlation coefficients (respectively, rm = 0.620 and 0.342). Interestingly, no significant correlations were observed between the sum of SFAs and triglycerides in either age group.

### FFA responses are influenced by the abundance and saturation type of the corresponding FA in yoghurt

**Fig. 6:**
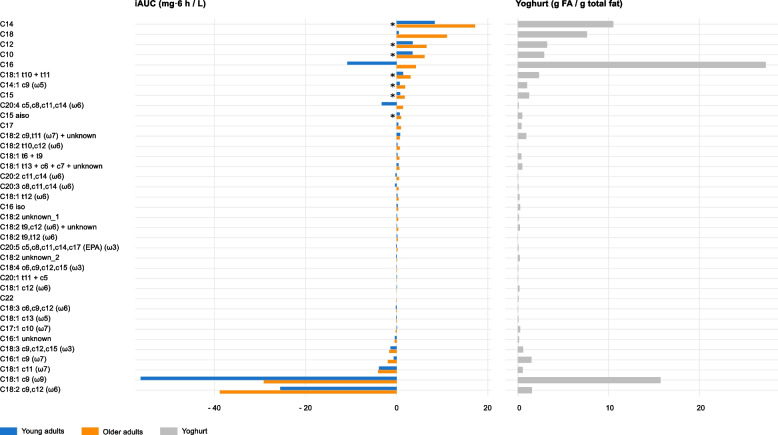
Yoghurt FA concentrations and their corresponding FFA postprandial responses. Blue and orange bars represent the median FFA iAUCs following yoghurt challenge in young and older adults, respectively. Grey bars represent the median FA concentrations in yoghurt. Values on the x-axis are displayed as iAUC (mg·6 h/L) for FFAs and as FA concentrations (g/100 g total fat) for yoghurt. Lipids are sorted in descending order based on the FFA iAUC of older adults. * iAUCs are significantly different between age groups, as assessed by the Mann–Whitney U test (FDR < 0.05). Data for the interquartile ranges, reflecting the variability in FFA iAUC, are provided in Supplementary Table 4. Some FFAs, such as C16 and C20:4 c5,c8,c11,c14 (ω6), presented particularly large variability. FA, fatty acid; FDR, false discovery rate; FFA, free fatty acid; iAUC, incremental area under the curve

The postprandial FFA responses were strongly influenced by the abundance of FAs in yoghurt (Fig. 6). FAs present at high concentrations in yoghurt, particularly saturated species with chain lengths between 10 and 18 carbons, generally produced positive iAUC values. Other highly abundant yoghurt FAs that elicited positive iAUC values were mostly unsaturated species containing trans bonds, such as C18:1 t10 + t11, C18:2 c9,t11 (ω7), C18:2 t10,c12 (ω6), and C18:1 t13 + c6 + c7. In contrast, abundant yoghurt FAs that elicited negative iAUC values were exclusively unsaturated and contained only cis bonds, with C18:1 c8 (ω9) and C18:2 c9,c12 (ω6) showing particularly negative iAUC values.

## Discussion

This study examined the metabolic effects of dairy fermentation and age-related differences in postprandial responses, with a major focus on glucose and lipid metabolism. Single acute consumption of yoghurt resulted in significantly lower postprandial glucose and insulin concentrations compared with milk. In contrast, triglyceride responses were primarily influenced by age, with older adults exhibiting higher postprandial concentrations and a delayed return to baseline levels, independent of the dairy product. Interestingly, age-related differences in triglycerides were observed only during the postprandial phase, whereas differences in FFA classes (SFA, MUFA and PUFA) and individual FFAs were already detectable in the fasting state and became more pronounced postprandially. In older adults, triglyceride and FFA responses to yoghurt were correlated, with age-related differences particularly evident in their heightened responses of saturated FFAs. Moreover, postprandial FFA responses were strongly influenced by the yoghurt’s FA composition. These findings contribute to a more nuanced understanding of the role of ageing in modulating postprandial metabolism and underscore the influence of specific food FA profiles on lipid handling across age groups.

Milk and yoghurt are recognised for their low carbohydrate content and are often grouped together in dietary guidelines with similar portion recommendations [32]. Nonetheless, yoghurt is well-documented to elicit a lower glycaemic index compared with milk, despite comparable insulin responses [33, 34]. Indeed, the recent qualified health claim approved by the United States Food and Drug Administration linking yoghurt consumption to a reduced risk of type 2 diabetes attributes the effect to yoghurt as a whole food rather than to individual nutrients [11]. In our study, carbohydrate content differed minimally between milk and yoghurt challenges (5.5 g lower per 600 mL yoghurt) [21], yet the observed reduction in glucose (68–73%) and insulin (8–22%) iCmax suggests that other mechanisms, possibly matrix-related factors, played a more prominent role. Structural and biochemical changes induced by fermentation, such as increased viscosity, gel formation and enhanced release of insulinotropic peptides from casein, can delay gastric emptying and enhance glucose uptake [8, 35–37], helping to explain the reduced peak glycaemic and insulinaemic responses with yoghurt, despite similar overall glucose and insulin exposures (iAUC). Attenuating postprandial excursions without altering total exposure may be particularly beneficial for older adults, as sharp glycaemic peaks are associated with oxidative stress and inflammation, contributing to endothelial dysfunction and cardiometabolic risk [38–40]. Overall, modifications of the food matrix in yoghurt appear to shape metabolic responses beyond nutrient composition, indicating that acute modulation of glucose dynamics could contribute to the improved glycaemic control and reduced cardiometabolic risk reported in epidemiological and intervention studies.

Yoghurt consumption has been associated with improvements in triglyceride levels in chronic settings, particularly when containing probiotic strains [9]; however, the evidence remains controversial [41, 42]. In our study, focusing on acute consumption, we did not observe any postprandial differences in triglyceride responses between milk and yoghurt. Fermentation of milk minimally altered total fat content [21], and while fatty acid profiles can differ depending on the starter culture used, few changes are observed in regular yoghurt [43]. In our study, triglyceride responses were driven primarily by age, and yoghurt did not attenuate the elevated responses observed in older adults. Instead, physiological changes associated with ageing – such as reduced lipoprotein lipase activity, delayed chylomicron clearance and diminished mitochondrial oxidative capacity [15, 44] – may not be readily overcome by acute and modest dietary modifications. Similarly to our findings, ingestion of a dairy product (whey protein) elicited higher GIP responses in older compared with younger individuals [45]. While GIP stimulates insulin and activates lipoprotein lipase [46], this compensatory mechanism appears insufficient to restore metabolic flexibility, as circulating lipid levels remained elevated in older adults despite higher GIP responses. If yoghurt improves lipid metabolism in the long term, the underlying mechanisms are likely unrelated to acute postprandial responses but may involve gradual improvements in insulin sensitivity [47]. Although no differences were observed in inflammatory markers such as TNF-α or IL-6 after a single acute intake by healthy individuals, yoghurt’s role in reducing oxidative stress and low-grade inflammation may still be relevant in chronic settings and warrants further investigation [48].

Impairments in lipid metabolism associated with ageing were further examined through detailed characterisation of FFA classes and individual FFAs, revealing distinct age-related patterns at both baseline and during the postprandial phase. At baseline, although the sums of SFAs, MUFAs and PUFAs differed between age groups, differences in individual FFAs were most pronounced among unsaturated fatty acids, with the majority being higher in older adults. Although studies directly comparing baseline individual FFA concentrations by age are scarce, Pararasa et al. [49] reported elevated plasma levels of several long-chain fatty acids in healthy men over 50 compared with younger counterparts. However, differences in FFA isomer configuration were not reported, limiting direct comparability. Mechanistically, age-related declines in delta-6 desaturase [50] and stearoyl-CoA desaturase [51] activity may contribute to the accumulation of long-chain polyunsaturated fatty acids and MUFAs, respectively. The cardiometabolic relevance of elevated FFAs, particularly SFAs, remains uncertain. A meta-analysis by Li et al. [52] associated increased cardiovascular risk primarily with even-chain SFAs, whereas odd-chain and very-long-chain SFAs were inversely associated. In contrast, Borges et al. [53] reported stronger associations for MUFAs, particularly palmitoleic and oleic acids, with no significant associations for SFAs. These inconsistencies suggest that individual fatty acid species, rather than broad saturation classes, may exert divergent metabolic effects.

In the postprandial state, significant age effects were evident, with elevated FFA concentrations persisting for up to six hours following the yoghurt challenge. These sustained elevations in older adults correlated strongly with triglyceride concentrations, reflecting their impaired return to baseline lipid levels compared with younger individuals. Although FFAs and triglycerides follow distinct physiological trajectories – FFAs typically declining initially due to insulin-mediated suppression of lipolysis and triglycerides gradually increasing – the stronger correlation observed in older adults may reflect reduced metabolic flexibility. Impaired insulin action in this context could drive concurrent elevations in both lipid classes [54, 55]. Additionally, the greater amplitude and variability of postprandial changes in older adults, evident already from two hour postprandially onwards, likely accentuated the statistical association between FFAs and triglycerides. Notably, correlations were the highest among individual saturated FFAs, as opposed to the sum of SFAs, all of which exhibited greater excursions and more pronounced age effects than unsaturated species. The accumulation of saturated FFAs may reflect attenuated insulin-mediated suppression of lipolysis combined with slower clearance kinetics, whereas unsaturated FFAs are more efficiently cleared due to their higher responsiveness to insulin regulation [55, 56]. Thus, detailed postprandial profiling of FFAs may serve as a sensitive marker of metabolic inflexibility, highlighting potential biomarkers relevant to age-associated cardiometabolic risk.

Postprandial FFA responses were also markedly influenced by the composition of the ingested dairy fat. FAs highly concentrated in yoghurt, particularly saturated species with chain lengths between 10 and 18, tended to produce the largest positive iAUC values in both age groups, consistent with previous evidence that plasma FFA profiles reflect the fatty acid composition of ingested dairy fat [57]. However, not all abundant yoghurt-derived FAs followed this pattern. Specifically, C18:1 c9 (ω9; oleic acid) and C18:2 c9,c12 (ω6; linoleic acid) displayed pronounced negative iAUC values. As described previously, unsaturated species are cleared more rapidly and respond more strongly to insulin-mediated suppression, accounting for their distinct postprandial kinetics compared with saturated FFAs. Moreover, structural features such as the presence of trans bonds influence oxidative stability and clearance. Indeed, trans unsaturated fatty acids share structural similarities with SFAs and exhibit slower oxidation than their cis counterparts [58]. Despite similarities between age groups in saturated FFAs baseline concentrations, older adults showed exaggerated postprandial excursions, reaffirming the heightened metabolic vulnerability under nutritional overload. Nevertheless, structural characteristics, particularly saturation and isomer configuration, remain key determinants shaping postprandial lipid responses and informing tailored dietary strategies.

The combined effects of lipid metabolism, metabolic flexibility and dairy fat composition were reflected in the distinct patterns of individual postprandial FFA responses. One cluster, comprising medium-chain saturated FFAs (C10 and C12), showed rapid postprandial increases, consistent with their efficient absorption and direct hepatic metabolism. Another cluster, enriched in FFAs of strong dairy fat origin (e.g., C15, C17, and C18:2 t10,c12), exhibited steadily rising and sustained elevations, particularly in older adults, suggesting coordinated digestion and incorporation into circulating lipoproteins. In contrast, a third cluster of structurally diverse lipids, including branched-chain and trans-fatty acids, displayed more variable trajectories with delayed peaks. These divergent kinetic signatures point to structural and metabolic determinants that differentially govern lipid handling, reinforcing characteristic patterns of postprandial lipid dynamics.

This study presents important strengths. The population was well characterised and both the restriction and challenge phases were conducted under highly standardised conditions. Rigorous control of the background diet helped minimise confounding from habitual intake and ensured consistency in metabolic conditions before the test days. Additionally, detailed profiling of 37 individual FFAs offers a unique and granular perspective on postprandial lipid metabolism. While interest in postprandial responses of established biochemical markers is strong, investigations of individual FFA kinetics, particularly in the context of ageing, remain rare. By evaluating individual FFAs alongside conventional biochemical markers, we were able to achieve a more integrated assessment of age-related differences in postprandial metabolism. The inclusion of this detailed lipid analysis not only underscores the novelty of our approach but also enhances the relevance of the findings.

However, certain limitations must be acknowledged. First, the study included a relatively small sample size, as it was originally designed to identify dietary biomarkers of milk and yoghurt consumption rather than to investigate age-related metabolic differences. This may have reduced statistical power to detect subtle effects, particularly increasing the risk of type II errors for outcomes with higher variability, such as inflammatory markers and incretin hormones. Second, the sample consisted exclusively of clinically healthy men, which limits the generalisability of findings to women and individuals with metabolic disorders. At the same time, this design choice may have reduced inter-individual variability, potentially enhancing sensitivity to detect mechanistic differences in postprandial responses. Third, the acute nature of the intervention, although suitable for assessing immediate metabolic responses, it does not allow for conclusions about long-term adaptations or disease risk. Forth, while the 600 mL dairy dose was appropriate for eliciting postprandial effects, it exceeds typical consumption patterns, limiting translation of findings to real-life dietary habits. Fifth, while key biochemical markers were measured, the panel of gastrointestinal hormones and inflammatory mediators was restricted, potentially overlooking other pathways relevant to postprandial regulation. Similarly, including body composition measurements (e.g., fat mass, lean mass) could have further enhanced the interpretability of the results. Furthermore, while detailed profiling of individual FFAs was performed, interpretation is constrained by the inability to distinguish endogenous release from dietary contributions in the postprandial state. Finally, FFA concentrations were measured only after the yoghurt challenge, precluding direct comparisons with milk and limiting the assessment of age × product interactions at the level of individual fatty acids.

In conclusion, our findings support growing evidence that yoghurt beneficially modulates glycaemic responses – an effect that, in acute settings, appears to be driven more by the food matrix than by nutrient composition alone. Conversely, the slower return of triglycerides and FFAs to baseline levels in older adults highlights the persistent metabolic challenges associated with ageing, emphasising the need to specifically target lipid metabolism in dietary strategies to reduce cardiometabolic risk for this population. Detailed profiling of individual postprandial FFAs could help uncover subtle metabolic dysregulations and impaired lipid handling, offering potential biomarkers for assessing metabolic flexibility, particularly in older adults. Future research should further examine their utility as early indicators of age-related metabolic decline to inform personalised strategies across the lifespan. Implementing nutritional strategies that target fat metabolism regulation would require investigating the postprandial FFA response to selected foods and meals with varying lipid profiles.

## Supplementary Information

Supplementary Material 1.

## Data Availability

Fatty acid data have been deposited at [10.5281/zenodo.15310288] and will be made available upon request. Any additional data described in the manuscript will be provided by the corresponding author upon reasonable request.

## Abbreviations

BMI: Body mass index
FDR: False discovery rate
FA: Fatty acid
FFA: Free fatty acid
GIP: Glucose-dependent insulinotropic polypeptide
iAUC: Incremental area under the curve
iCmax: Incremental maximum concentration
IL-6: Interleukin-6
iTmax: Time at incremental maximum concentration
MUFA: Monounsaturated fatty acid
OA: Older adults
PUFA: Polyunsaturated fatty acid
SFA,: Saturated fatty acid
TNF-α: Tumour necrosis factor-alpha
YA: Young adults
