# Supplementary material for: Postprandial metabolic effects of milk and yoghurt in young and older adults
Source: Genes Nutr. 2025 Oct 13;20:19. doi: 10.1186/s12263-025-00780-x (PMC12519632; doi:10.1186/s12263-025-00780-x)
Supplement: Supplementary file 1 — Supplementary Material 1. [file 12263_2025_780_MOESM1_ESM.pdf]

Postprandial Metabolic Effects of Milk and Yoghurt in Young and Older Adults

Elaine Hillesheim [1], Gaïa Lépine [2], Patrick Neuhaus [1], Kathryn J. Burton-Pimentel [1], Jinyoung Kim [1,2,3], Ulrich Bütikofer [1], Charlotte Fleuti [1], Corinne Marmonier [3], Dominique Dardevet [2], Sergio Polakof [2], Guy Vergères [1*]

[1] Agroscope, Liebefeld, CH-3003 Bern, Switzerland

[2] Unité de Nutrition Humaine (UNH), INRAE, Université Clermont Auvergne, F-63000 Clermont-Ferrand, France

[3] CNIEL, 42 Rue de Châteaudun, F-75009 Paris, France

*** Corresponding author:** guy.vergeres@agroscope.admin.ch

Table of contents

**Table S1**	Participants' characteristics at baseline by study period ………………….	3
**Table S2**	Postprandial responses of clinical chemistry markers to milk and yoghurt intervention, by age group …………………...…………………………	4
**Table S3**	Free fatty acids concentrations at baseline by age groups …………………	6
**Table S4**	Free fatty acids responses to the yoghurt intervention, by age group …...…	7
**Table S5**	Free fatty acids included in the analyses and their corresponding CAS registry numbers and IUPAC names ……………………….…….……	9
**Figure S1**	Temporal comparison of postprandial glucose and insulin responses between test products and within age groups …………………………...…	11
**Figure S2**	Temporal comparison of postprandial triglycerides and GIP responses between age groups and within test products ……………………………	12
**Figure S3**	Postprandial TNF-α, IL-6 and ghrelin responses to milk and yoghurt challenges ……………………………………………………………..…..	13
**Figure S4**	Temporal comparison of postprandial FFA responses between age groups to yoghurt challenge ……………………………………………………..…	14
**Figure S5**	Repeated measures correlations between postprandial triglycerides and FFA responses to yoghurt challenge ………………………………………	15
**Figure S6**	Absolute postprandial responses of biochemical markers to milk and yoghurt challenges ……..………………….……………………………..	16
**Figure S7**	Absolute postprandial responses of FFA classes by age groups to yoghurt challenge ………………………..……………………..…………..	17

Table S1. Participants' characteristics at baseline by study period

	Test day 1 (n = 28)	Test day 2 (n = 28)	P-value
BMI (kg/m^2)	24.5 (22.5, 26.1)	24.4 (22.4, 26.0)	0.001
Weight (kg)	75.3 (68.4, 82.5)	74.8 (67.8, 80.8)	0.001
Glucose (mmol/L)	4.91 (4.69, 5.25)	5.00 (4.70, 5.22)	0.473
Insulin (pmol/L)	23.9 (16.1, 31.7)	23.0 (16.1, 33.4)	0.811
Triglycerides (mmol/L)	0.84 (0.66, 1.08)	0.81 (0.69, 0.96)	0.964
TNF-α (pg/mL)	7.04 (5.71, 10.25)	6.02 (5.28, 7.85)	0.136
IL-6 (pg/mL)	0.98 (0.74, 1.40) *	1.18 (0.88, 1.93)	0.264
GIP (pg/L)	0.067 (0.054, 0.083)	0.076 (0.052, 0.112)	0.322
Ghrelin (pg/L)	3.66 (3.06, 5.20)	4.39 (2.64, 5.96)	0.276

Values are medians and interquartile ranges. Differences between test days were assessed using the Wilcoxon signed-rank test. * One missing value resulted in 27 measurements. BMI, body mass index; GIP, glucose-dependent insulinotropic polypeptide; IL-6, interleukin-6; TNF-α, tumour necrosis factor-alpha.

Table S2. Postprandial responses of clinical chemistry markers to milk and yoghurt intervention, by age group

		Young adults		Older adults		Time effect	Age effect	Product effect	Time *x* age	Time *x* product	Age *x* product	Age *x* product *x* time
		**Milk (n = 14)**	**Yoghurt (n = 14)**	**Milk (n = 14)**	**Yoghurt (n = 14)**							
Glucose (mmol/L)	iAUC	-1.16 (-2.60, 0.91)	-1.61 (-2.05, -1.14) d#	-0.67 (-1.55, 0.60)	-0.72 (-1.72, 0.46) d#	**<0.001**	0.240	**0.030**	**0.028**	**0.001**	0.163	0.613
	iCmax	1.03 (0.54, 1.39) a*	0.28 (0.15, 0.61) a*	1.16 (0.30, 1.59) b*	0.37 (0.19, 0.59) b*							
	iTmax	0.50 (0.50, 0.50)	0.50 (0.50, 0.50)	0.50 (0.50, 0.50)	0.50 (0.50, 1.38)							
Insulin (pmol/L)	iAUC	129 (65, 233) a#	113 (96, 142) a#	137 (109, 214)	123 (107, 142)	**<0.001**	0.255	**0.011**	**0.001**	**<0.001**	0.462	0.736
	iCmax	138 (102, 261) a#	108 (99, 159) a#	106 (93, 182) b*	98 (76, 111) b*							
	iTmax	0.50 (0.50, 0.50)	0.50 (0.50, 0.88)	0.50 (0.50, 0.50)	0.50 (0.50, 0.88)							
Triglycerides (mmol/L)	iAUC	0.17 (-0.23, 1.39) c#	0.54 (-0.14, 1.28) d*	1.26 (0.85, 1.53) c#	1.09 (0.84, 1.60) d*	**<0.001**	**0.011**	0.298	**<0.001**	**<0.001**	0.548	0.255
	iCmax	0.19 (0.12, 0.53) c#	0.29 (0.15, 0.48)	0.43 (0.34, 0.57) c#	0.41 (0.34, 0.51)							
	iTmax	1.50 (1.00, 3.00) c*	2.00 (1.63, 3.00) d*	3.50 (3.00, 4.00) c*	3.00 (3.00, 3.00) d*							
TNF-α (pg/mL)	iAUC	-1.23 (-9.97, 1.13) c*	-1.17 (-4.49, 0.65)	1.35 (-0.24, 3.17) c*	0.44 (-1.17, 4.44)	0.140	**0.009**	0.666	0.071	0.860	0.648	0.077
	iCmax	0.78 (-0.49, 1.99) c#	0.53 (-1.03, 1.86)	2.35 (1.15, 4.45) c#	2.01 (0.57, 3.34)							
	iTmax	0.75 (0.50, 2.50)	0.75 (0.50, 1.00) d*	0.50 (0.50, 2.50)	2.00 (1.00, 3.00) d*							
IL-6 (pg/mL)	iAUC	0.22 (-0.44, 0.87)	0.24 (-0.37, 0.71)	0.17 (-0.32, 0.95)	0.11 (-1.18, 0.63)	**<0.001**	0.860	0.585	0.754	0.505	0.278	0.628
	iCmax	0.55 (-0.08, 0.95)	0.34 (0.16, 0.71)	0.24 (0.02, 0.56)	0.30 (0.14, 0.65)							
	iTmax	3.00 (0.63, 3.00)	3.00 (1.50, 3.00)	3.00 (1.00, 3.00)	3.00 (3.00, 3.00)							
GIP (pg/L)	iAUC	0.35 (0.23, 0.52) c#	0.50 (0.34, 0.59)	0.51 (0.46, 0.54) #	0.62 (0.45, 0.78)	**<0.001**	**0.014**	0.246	**0.023**	**<0.001**	0.584	0.337
	iCmax	0.28 (0.19, 0.32)	0.29 (0.23, 0.38)	0.26 (0.23, 0.37)	0.34 (0.24, 0.43)							
	iTmax	0.50 (0.50, 0.50) c#	1.00 (0.50, 1.00)	0.75 (0.50, 3.00) c#	1.00 (1.00, 1.00)							
Ghrelin (pg/L)	iAUC	-1.49 (-3.37, -0.39)	-1.42 (-3.47, -0.08)	-1.54 (-3.23, -0.60)	-1.66 (-2.79, -0.46)	**<0.001**	0.944	0.147	0.181	**0.029**	0.877	0.881
	iCmax	-0.02 (-0.57, 1.85)	0.12 (-0.34, 0.70)	-0.18 (-0.54, 0.46)	-0.14 (-0.78, 0.85)							
	iTmax	3.00 (1.00, 3.00)	1.75 (0.50, 3.00)	2.00 (1.00, 3.00)	0.50 (0.50, 3.00)							

Values are medians and interquartile ranges. Postprandial responses for glucose, insulin and triglycerides were assessed from 0-6 h, while postprandial responses for TNF-α, IL-6, GIP and ghrelin were assessed from 0-3 h. Intervention effects were assessed using the Wald test from the Nonparametric Analysis of Longitudinal Data in Factorial Experiments. Paired comparisons within age groups and between product challenges were performed using the Wilcoxon signed-rank test and are indicated by the letters 'a' and 'b'. Unpaired comparisons between age groups for the same product challenge were performed using the Mann-Whitney U test and are indicated by the letters 'c' and 'd'. Within the same row, values marked with 'a' differ from other also marked with 'a', values marked with 'b' differ from other also marked with 'b', and so on. # $p < 0.10$, * $p < 0.05$. GIP, glucose-dependent insulinotropic polypeptide; iAUC, incremental area under the curve; iCmax, incremental maximum concentration; iTmax, time in hours at incremental maximum concentration; IL-6, interleukin-6; TNF-α, tumour necrosis factor-alpha.

Table S3. Free fatty acids concentrations at baseline by age groups

Free fatty acid	Young adults (n = 14)	Older adults (n = 14)	FDR
Sum SFA	88.5 (72.7, 98.1)	122.6 (100.9, 139.8)	**0.020**
Sum MUFA	64.0 (47.5, 73.7)	83.6 (72.2, 99.1)	**0.033**
Sum PUFA	62.8 (58.7, 72.1)	83.8 (69.2, 101.1)	**0.018**
C10	0.05 (0, 0.07)	0 (0, 0)	**0.028**
C12	0.35 (0.20, 0.52)	0.31 (0.27, 0.45)	0.960
C14	2.02 (1.64, 2.58)	2.64 (2.16, 3.86)	0.126
C14:1 c9 (ω5)	0.20 (0.14, 0.30)	0.19 (0.16, 0.24)	0.871
C15	0.55 (0.44, 0.62)	0.69 (0.57, 0.83)	0.144
C15 aiso	0.17 (0.11, 0.20)	0.09 (0.05, 0.14)	0.126
C16	60.3 (47.7, 67.9)	83.9 (69.2, 93.4)	0.120
C16 iso	0.10 (0.09, 0.14)	0.14 (0.09, 0.19)	0.335
C16:1 c9 (ω7)	2.39 (1.67, 4.03)	4.91 (3.78, 5.96)	**0.033**
C16:1 unknown	0.64 (0.53, 0.71)	0.95 (0.55, 1.18)	0.245
C17	0.71 (0.55, 0.76)	0.80 (0.44, 1.00)	0.626
C17:1 c10 (ω7)	0.38 (0.33, 0.45)	0.41 (0.37, 0.48)	0.614
C18	24.6 (22.1, 26.3)	34.7 (27.4, 39.7)	0.074
C18:1 c11 (ω7)	4.48 (4.04, 5.11)	6.87 (5.44, 7.29)	**0.040**
C18:1 c12 (ω6)	0.29 (0.21, 0.35)	0.41 (0.30, 0.49)	0.112
C18:1 c13 (ω5)	0.18 (0.14, 0.21)	0.17 (0.12, 0.21)	0.770
C18:1 c9 (ω9)	52.4 (39.5, 62.9)	69.8 (59.1, 80.4)	0.149
C18:1 t10 + t11	0.28 (0.16, 0.39)	0.54 (0.27, 0.61)	0.126
C18:1 t12 (ω6)	0.25 (0.21, 0.30)	0.26 (0.19, 0.29)	1.000
C18:1 t13 + c6 + c7 + unknown	0.25 (0.18, 0.34)	0.30 (0.26, 0.33)	0.335
C18:1 t6 + t9	0.41 (0.30, 0.47)	0.43 (0.31, 0.57)	0.614
C18:2 c9,c12 (ω6)	39.8 (37.4, 44.1)	58.1 (46.9, 71.8)	**0.033**
C18:2 c9,t11 (ω7) + unknown	0.20 (0.11, 0.30)	0.21 (0.14, 0.40)	0.858
C18:2 t10,c12 (ω6)	0.05 (0.03, 0.06)	0.07 (0.04, 0.11)	0.384
C18:2 t9,c12 (ω6) + unknown	0.11 (0.08, 0.13)	0.11 (0.09, 0.13)	0.754
C18:2 t9,t12 (ω6)	0.12 (0.06, 0.15)	0.23 (0.18, 0.30)	0.058
C18:2 unknown_1	0.26 (0.20, 0.35)	0.32 (0.24, 0.37)	0.495
C18:2 unknown_2	0.52 (0.47, 0.64)	0.30 (0.16, 0.49)	0.245
C18:3 c6,c9,c12 (ω6)	0.40 (0.33, 0.49)	0.60 (0.34, 0.88)	0.335
C18:3 c9,c12,c15 (ω3)	1.56 (1.20, 1.96)	2.61 (2.12, 3.15)	**0.028**
C18:4 c6,c9,c12,c15 (ω3)	0.10 (0.09, 0.14)	0.08 (0.07, 0.11)	0.384
C20:1 t11 + c5	0.17 (0.09, 0.29)	0.14 (0.09, 0.21)	0.804
C20:2 c11,c14 (ω6)	0.55 (0.46, 0.64)	0.68 (0.55, 0.87)	0.245
C20:3 c8,c11,c14 (ω6)	2.85 (2.28, 3.38)	2.90 (1.58, 3.27)	0.626
C20:4 c5,c8,c11,c14 (ω6)	15.9 (13.0, 17.9)	15.9 (11.6, 21.8)	1.000
C20:5 c5,c8,c11,c14,c17 (EPA) (ω3)	1.31 (0.83, 1.70)	1.60 (0.98, 1.91)	0.495
C22	0.18 (0.12, 0.24)	0.25 (0.15, 0.36)	0.335

Values are medians and interquartile ranges. Concentrations are mg/L. Differences between age groups were assessed using the Mann-Whitney U test and corrected for multiple testing using FDR.

Table S4. Free fatty acids responses to the yoghurt intervention, by age group

**Free fatty acid**	**Young adults (n = 14)**	**Older adults (n = 14)**	**iAUC ***	**nparLD §**		
				**Time effect**	**Age effect**	**Time *x* Age**
Sum SFA **	10.3 (-14.7, 55.6)	65.7 (46.8, 148.2)	**0.023**	**<0.001**	**0.021**	**0.004**
Sum MUFA **	-49.6 (-66.1, -10.3)	0.7 (-82.2, 29.5)	0.448	**<0.001**	0.440	0.109
Sum PUFA	-29.4 (-65.6, -10.2)	-22.9 (-45.4, 1.4)	0.765	**<0.001**	0.645	**0.033**
C14 **	8.33 (6.12, 9.83)	17.2 (14.7, 20.2)	**0.010**	**<0.001**	**<0.001**	**<0.001**
C18	0.42 (-14.8, 11.1)	11.0 (-3.20, 31.7)	0.360	**<0.001**	0.401	0.055
C12 **	3.51 (2.19, 3.94)	6.53 (5.76, 6.91)	**0.010**	**<0.001**	**<0.001**	**0.001**
C10 **	3.48 (2.84, 4.46)	6.11 (4.17, 7.57)	**0.037**	**<0.001**	**0.006**	**0.001**
C16	-10.8 (-33.3, 10.6)	4.23 (-7.93, 63.4)	0.286	**<0.001**	0.261	**0.018**
C18:1 t10 + t11	1.42 (1.33, 2.55)	3.03 (2.23, 4.44)	**0.039**	**<0.001**	**0.010**	**0.001**
C14:1 c9 (ω5) **	0.65 (0.48, 1.06)	1.83 (1.39, 2.14)	**0.015**	**<0.001**	**<0.001**	**<0.001**
C15 **	0.76 (0.62, 1.07)	1.75 (1.18, 2.44)	**0.026**	**<0.001**	**0.003**	**<0.001**
C20:4 c5,c8,c11,c14 (ω6)	-3.27 (-12.4, 3.50)	1.39 (-4.02, 5.07)	0.652	0.124	0.458	0.238
C15 aiso	0.66 (0.41, 0.74)	0.97 (0.79, 1.28)	0.057	**<0.001**	**0.043**	**0.002**
C17	0.34 (0.06, 0.67)	0.94 (0.58, 1.34)	0.083	**<0.001**	0.134	0.165
C18:2 c9,t11 (ω7) + unknown	0.73 (0.55, 0.93)	0.68 (0.27, 1.83)	0.971	**<0.001**	0.946	0.165
C18:2 t10,c12 (ω6)	0.18 (0.09, 0.30)	0.65 (0.16, 0.94)	0.126	**<0.001**	0.167	0.237
C18:1 t6 + t9	0.17 (0.05, 0.31)	0.58 (0.16, 1.10)	0.135	**<0.001**	0.151	0.165
C18:1 t13 + c6 + c7 + unknown	0.40 (0.16, 0.58)	0.56 (0.12, 0.81)	0.845	**<0.001**	0.813	0.661
C20:2 c11,c14 (ω6)	-0.25 (-0.56, -0.07)	0.49 (-0.41, 1.35)	0.262	**0.001**	0.607	0.308
C20:3 c8,c11,c14 (ω6)	-0.35 (-1.85, 0.16)	0.42 (-0.11, 1.24)	0.286	**0.033**	0.239	0.050
C18:1 t12 (ω6)	0.17 (0.05, 0.35)	0.40 (0.10, 0.56)	0.429	**<0.001**	0.513	0.751
C16 iso	0.21 (0.15, 0.25)	0.33 (0.22, 0.42)	0.286	**<0.001**	0.458	0.165
C18:2 unknown_1	0.11 (-0.25, 0.29)	0.31 (-0.12, 0.37)	0.429	**<0.001**	0.513	0.229

continue

Table S4. Free fatty acids responses to the yoghurt intervention, by age group *(continuation)*

**Free fatty acid**	**Young adults (n = 14)**	**Older adults (n = 14)**	**iAUC ***	**nparLD §**		
				**Time effect**	**Age effect**	**Time *x* age**
C18:2 t9,c12 (ω6) + unknown	0.03 (-0.07, 0.17)	0.30 (0.03, 0.88)	0.154	**<0.001**	0.167	0.352
C18:2 t9,t12 (ω6)	0.13 (-0.22, 0.34)	0.22 (-0.31, 0.63)	0.845	**0.001**	0.710	0.238
C20:5 c5,c8,c11,c14,c17 (EPA) (ω3)	-0.15 (-0.75, 0.16)	0.18 (-0.42, 1.02)	0.295	0.091	0.458	0.237
C18:2 unknown_2	-0.13 (-0.19, 0.05)	0.08 (-0.85, 0.36)	0.971	**<0.001**	0.946	0.059
C18:4 c6,c9,c12,c15 (ω3)	0.00 (-0.06, 0.08)	0.05 (-0.16, 0.20)	0.836	0.370	0.946	0.767
C20:1 t11 + c5	0.03 (-0.19, 0.38)	0.02 (-0.02, 0.16)	0.845	0.069	0.740	0.912
C18:1 c12 (ω6)	0.04 (-0.29, 0.20)	-0.01 (-0.42, 0.24)	0.971	**<0.001**	0.946	0.882
C22	0.00 (-0.20, 0.41)	-0.03 (-0.25, 0.29)	0.971	0.201	0.710	0.308
C18:3 c6,c9,c12 (ω6)	-0.19 (-0.29, 0.18)	-0.09 (-0.28, 0.20)	0.845	**<0.001**	0.710	0.109
C18:1 c13 (ω5)	-0.07 (-0.21, 0.05)	-0.09 (-0.19, 0.24)	0.836	**<0.001**	0.626	0.560
C17:1 c10 (ω7)	0.08 (-0.08, 0.33)	-0.21 (-0.50, 0.07)	0.286	**0.044**	0.498	0.064
C16:1 unknown	-0.38 (-0.71, -0.09)	-0.44 (-1.51, 0.20)	0.982	**<0.001**	0.659	0.912
C18:3 c9,c12,c15 (ω3)	-1.34 (-2.23, -0.21)	-1.63 (-3.38, 0.59)	0.845	**<0.001**	0.825	**0.034**
C16:1 c9 (ω7)	-0.65 (-4.25, 0.15)	-1.92 (-5.49, 3.15)	0.971	**<0.001**	0.946	**0.048**
C18:1 c11 (ω7)	-3.86 (-4.42, -0.95)	-4.09 (-7.83, -0.76)	0.971	**<0.001**	0.946	0.099
C18:1 c9 (ω9)	-56.2 (-65.4, -32.9)	-29.1 (-83.7, -4.83)	0.845	**<0.001**	0.698	0.111
C18:2 c9,c12 (ω6)	-25.5 (-48.6, -14.2)	-38.8 (-52.1, -14.6)	0.971	**<0.001**	0.967	0.064

Values are medians and interquartile ranges of the iAUC for postprandial free fatty acid responses, expressed as mg·6 h/L. Free fatty acids are sorted in descending order based on the iAUC of older adults. Differences in iAUC and iCmax between age groups were assessed using the Mann-Whitney U test and corrected for multiple testing using FDR. * FDR for comparing iAUC between age groups. ** iCmax values are significantly different between age groups. § Intervention effects were assessed using the Wald test from the Nonparametric Analysis of Longitudinal Data in Factorial Experiments (nparLD) and corrected for multiple testing, within effect types, using FDR. Statistical significance is set at FDR < 0.05. FDR, false discovery rate; iAUC, incremental area under the curve; iCmax, incremental maximum concentration.

Table S5. Free fatty acids included in the analyses and their corresponding CAS registry numbers and IUPAC names

Free fatty acid	CAS registry number	IUPAC name
C10	334-48-5	Decanoic acid
C12	143-07-7	Dodecanoic acid
C14	544-63-8	Tetradecanoic acid
C14:1 c9 (ω5)	544-64-9	(Z)-Tetradec-9-enoic acid
C15	1002-84-2	Pentadecanoic acid
C15 aiso	5502-94-3	12-Methyltetradecanoic acid
C16	67701-02-4	Hexadecanoic acid
C16 iso	4669-02-7	14-Methylpentadecanoic acid
C16:1 c9 (ω7)	2091-29-4	(Z)-Hexadec-9-enoic acid
C16:1 unknown	-	Hexadecenoic acid (unknown)
C17	63399-94-0	Heptadecanoic acid
C17:1 c10 (ω7)	29743-97-3	(Z)-Heptadec-10-enoic acid
C18	57-11-4	Octadecanoic acid
C18:1 c9 (ω9)	112-80-1	(Z)-Octadec-9-enoic acid
C18:1 c11 (ω7)	506-17-2	(Z)-Octadec-11-enoic acid
C18:1 c12 (ω6)	13126-37-9	(Z)-Octadec-12-enoic acid
C18:1 c13 (ω5)	13126-39-1	(Z)-Octadec-13-enoic acid
C18:1 t6 + t9	-	(E)-Octadec-6-enoic acid + (E)-Octadec-9-enoic acid
C18:1 t10 + t11	693-72-1	(E)-Octadec-10-enoic acid + (E)-Octadec-11-enoic acid
C18:1 t12 (ω6)	7378-88-3	(E)-Octadec-12-enoic acid
C18:1 t13 + c6 + c7 + unknown	-	(E)-Octadec-13-enoic acid + (Z)-Octadec-6-enoic acid + (Z)-Octadec-7-enoic acid + unknown
C18:2 c9,c12 (ω6)	60-33-3	(9Z,12Z)-Octadeca-9,12-dienoic acid

continue

Table S5. Free fatty acids included in the analyses and their corresponding CAS numbers and IUPAC names *(continuation)*

**Free fatty acid**	**CAS registry number**	**IUPAC name**
C18:2 c9,t11 (ω7) + unknown	2540-56-9	(9Z,11E)-Octadeca-9,11-dienoic acid + unknown
C18:2 t9,c12 (ω6) + unknown	2420-55-5	(9E,12Z)-Octadeca-9,12-dienoic acid + unknown
C18:2 t9,t12 (ω6)	506-21-8	(9E,12E)-Octadeca-9,12-dienoic acid
C18:2 t10,c12 (ω6)	2420-56-6	(10E,12Z)-Octadeca-10,12-dienoic acid
C18:2 unknown_1	-	Octadecadienoic acid (unknown 1)
C18:2 unknown_2	-	Octadecadienoic acid (unknown 2)
C18:3 c6,c9,c12 (ω6)	506-26-3	(6Z,9Z,12Z)-Octadeca-6,9,12-trienoic acid
C18:3 c9,c12,c15 (ω3)	463-40-1	(9Z,12Z,15Z)-Octadeca-9,12,15-trienoic acid
C18:4 c6,c9,c12,c15 (ω3)	20290-75-9	(6Z,9Z,12Z,15Z)-Octadeca-6,9,12,15-tetraenoic acid
C20:1 t11 + c5	2462-94-4, 7050-07-9	(E)-Icos-11-enoic acid + (Z)-Icos-5-enoic acid
C20:2 c11,c14 (ω6)	5598-38-9	(11Z,14Z)-Icosa-11,14-dienoic acid
C20:3 c8,c11,c14 (ω6)	1783-84-2	(8Z,11Z,14Z)-Icosa-8,11,14-trienoic acid
C20:4 c5,c8,c11,c14 (ω6)	506-32-1	(5Z,8Z,11Z,14Z)-Icosa-5,8,11,14-tetraenoic acid
C20:5 c5,c8,c11,c14,c17 (EPA) (ω3)	10417-94-4	(5Z,8Z,11Z,14Z,17Z)-Icosa-5,8,11,14,17-pentaenoic acid
C22	112-85-6	Docosanoic acid

CAS, Chemical Abstracts Service; IUPAC, International Union of Pure and Applied Chemistry

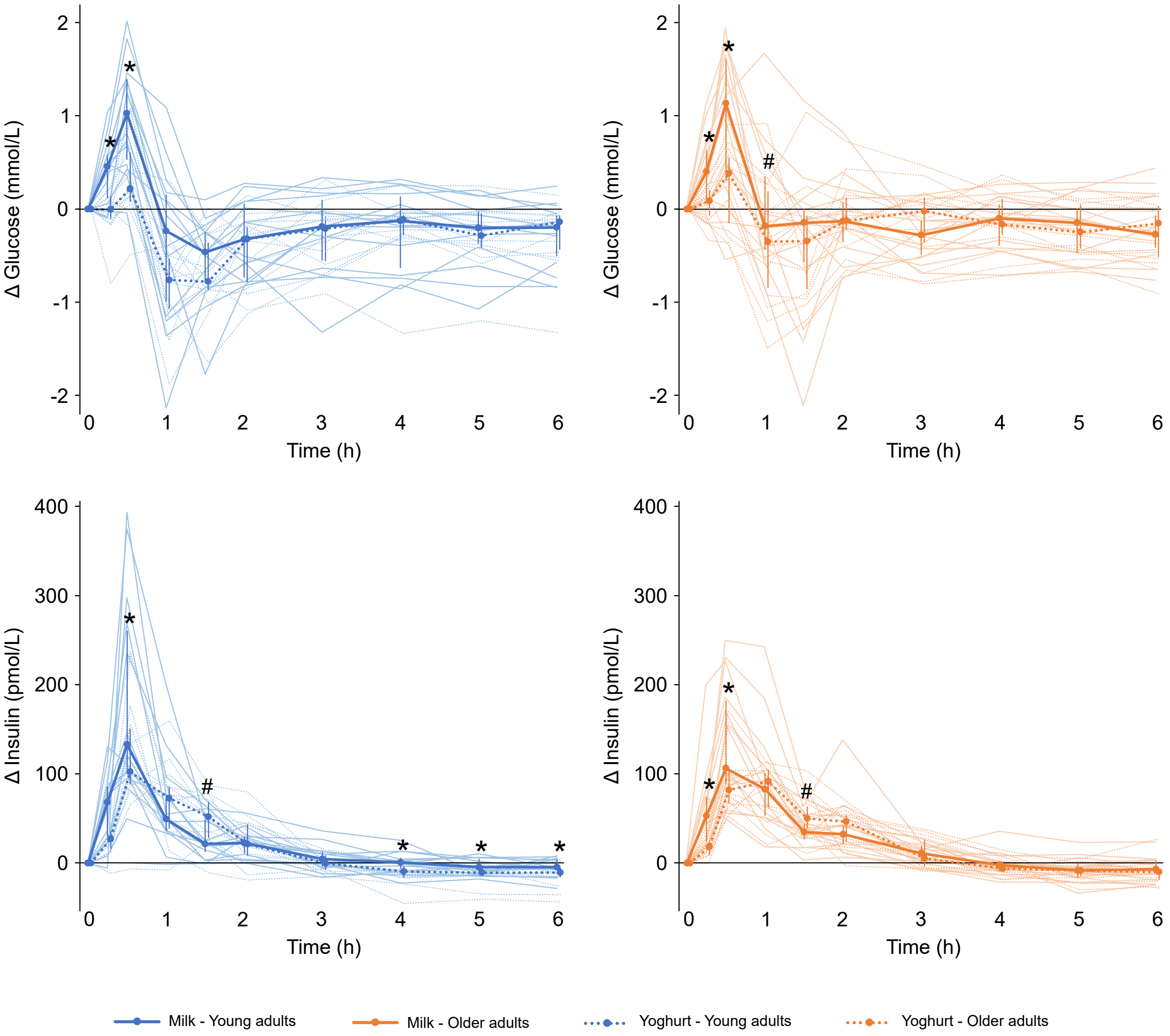

Figure S1. Temporal comparison of postprandial glucose and insulin responses between test products and within age groups. Line plots display postprandial responses as medians with interquartile ranges. The Wilcoxon signed-rank test was used to compare responses between test products within each time point. # $p < 0.10$, * $p < 0.05$.

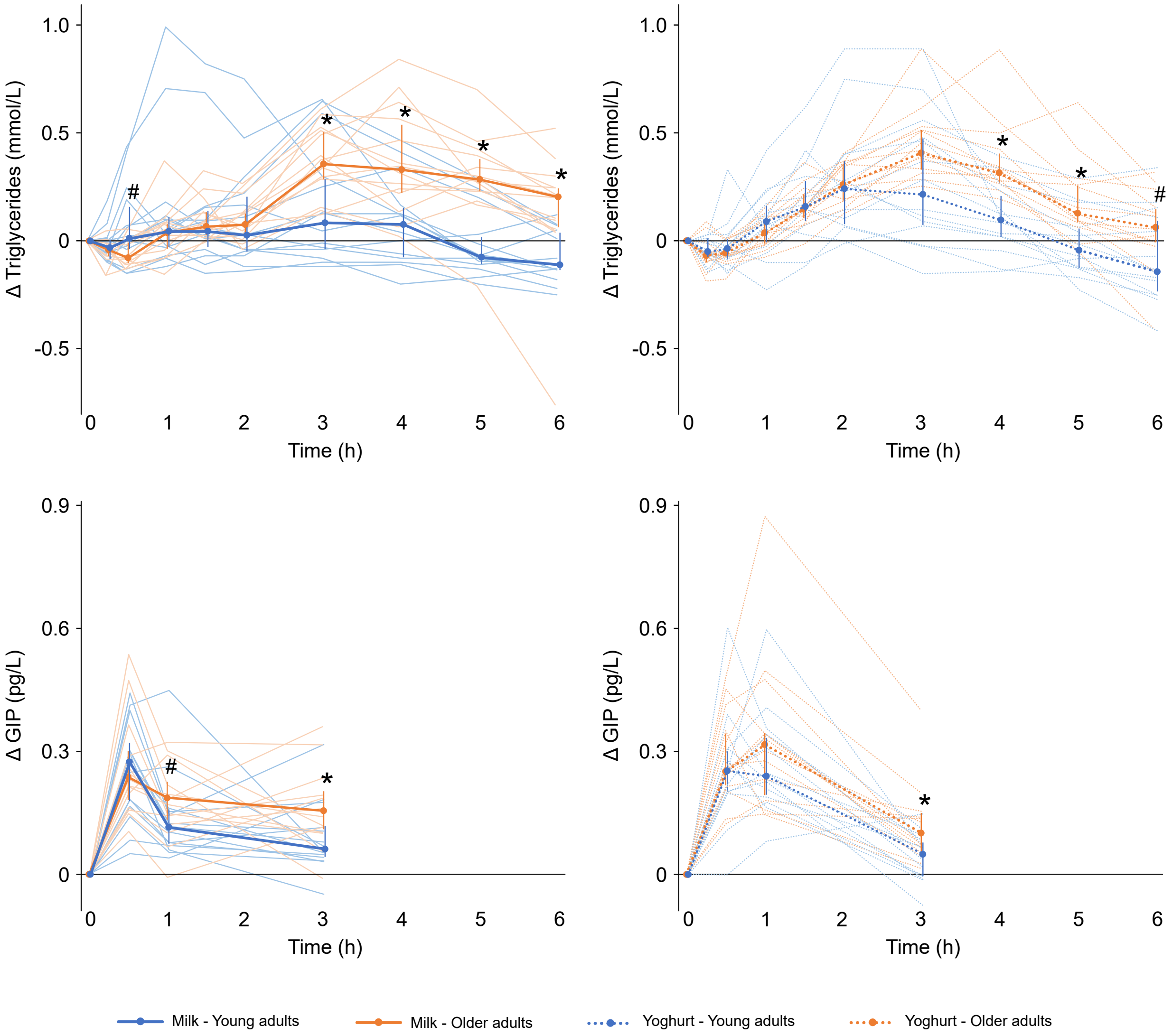

Figure S2. Temporal comparison of postprandial triglycerides and GIP responses between age groups and within test products. Line plots display postprandial responses as medians with interquartile ranges. The Mann-Whitney U test was used to compare responses between age groups within each time point. # $p < 0.10$, * $p < 0.05$. GIP, glucose-dependent insulinotropic polypeptide.

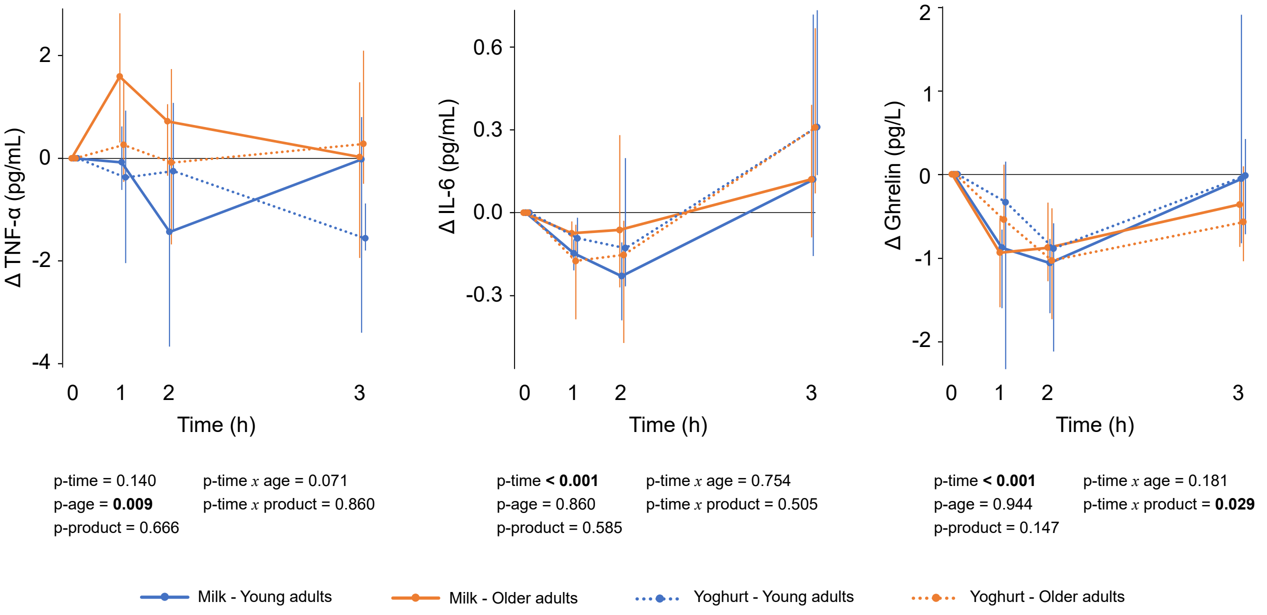

Figure S3. Postprandial TNF-α, IL-6 and ghrelin responses to milk and yoghurt challenges. Line plots display postprandial responses as medians with interquartile ranges. Intervention effects were assessed using the Wald test from the Nonparametric Analysis of Longitudinal Data in Factorial Experiments. All age × product and time × age × product interactions were non-significant ($p > 0.05$). Line plots of absolute postprandial responses are provided in Supplementary Figure S6. IL-6, interleukin-6; TNF-α, tumour necrosis factor-alpha.

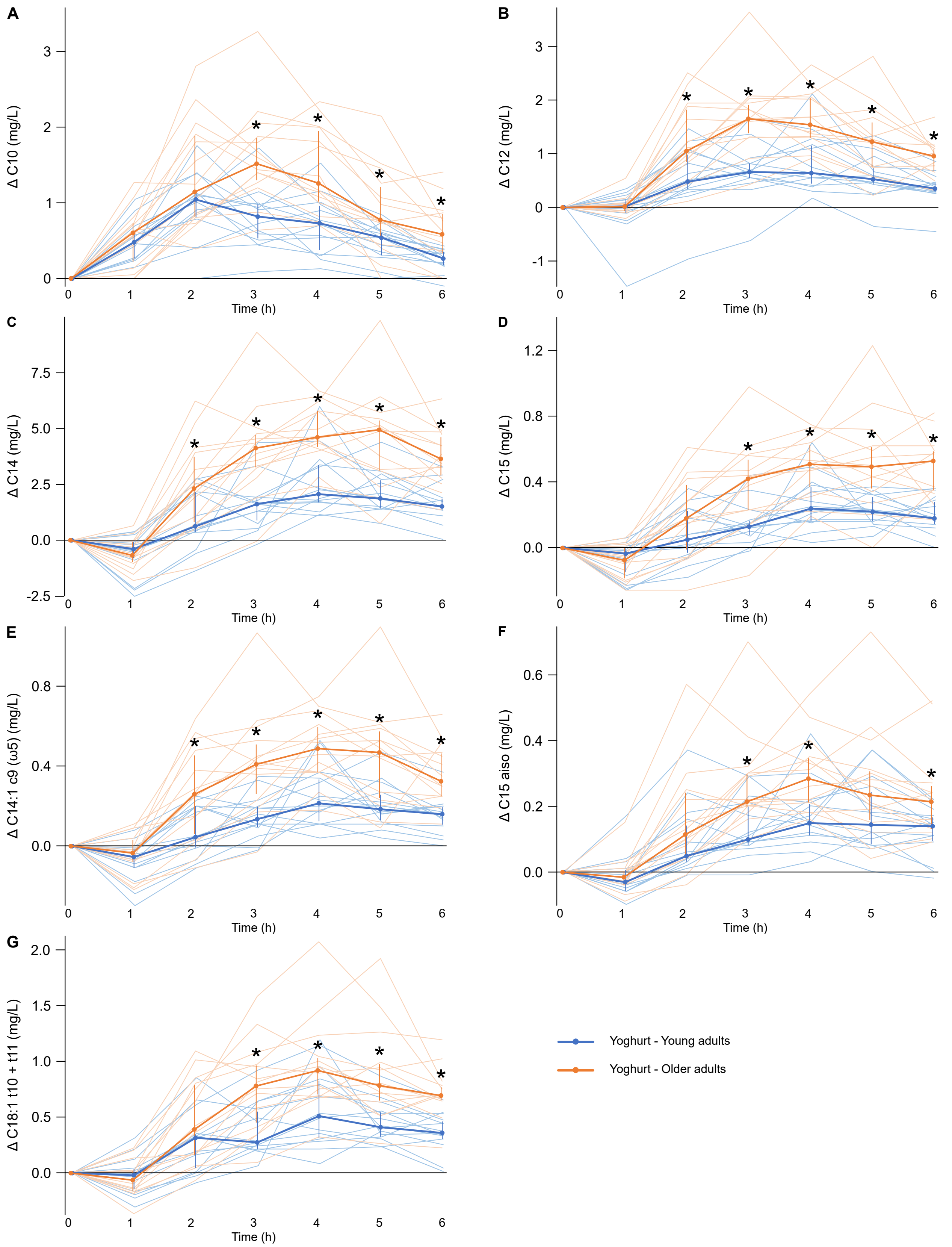

Figure S4. Temporal comparison of postprandial FFA responses between age groups to yoghurt intake. These FFA presented significant time, age and time × age effects in longitudinal analysis (FDR < 0.05). Line plots display postprandial responses as medians with interquartile ranges. The Mann-Whitney U test was used to compare responses between age groups within each time point. # $p < 0.10$, * $p < 0.05$. FDR, false discovery rate; FFA, free fatty acids.

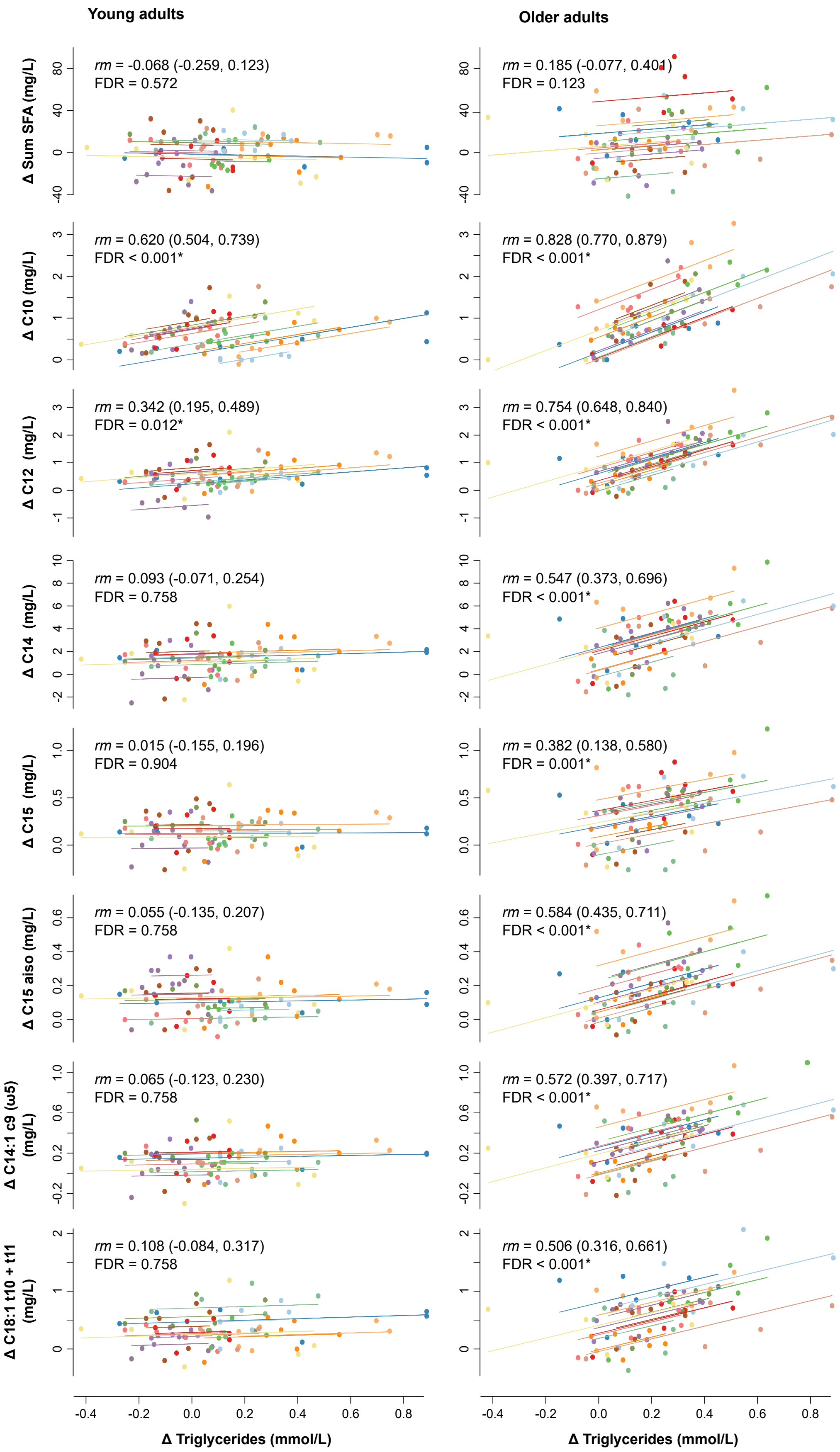

Figure S5. Repeated measures correlations between postprandial triglycerides and FFA responses to yoghurt challenge. Correlations were calculated using incremental concentrations of both triglycerides and FFAs across six time points. Within each age group, colours represent individual participants. FDR, false discovery rate; *rm*, repeated measures correlation coefficient with 95% confidence interval.

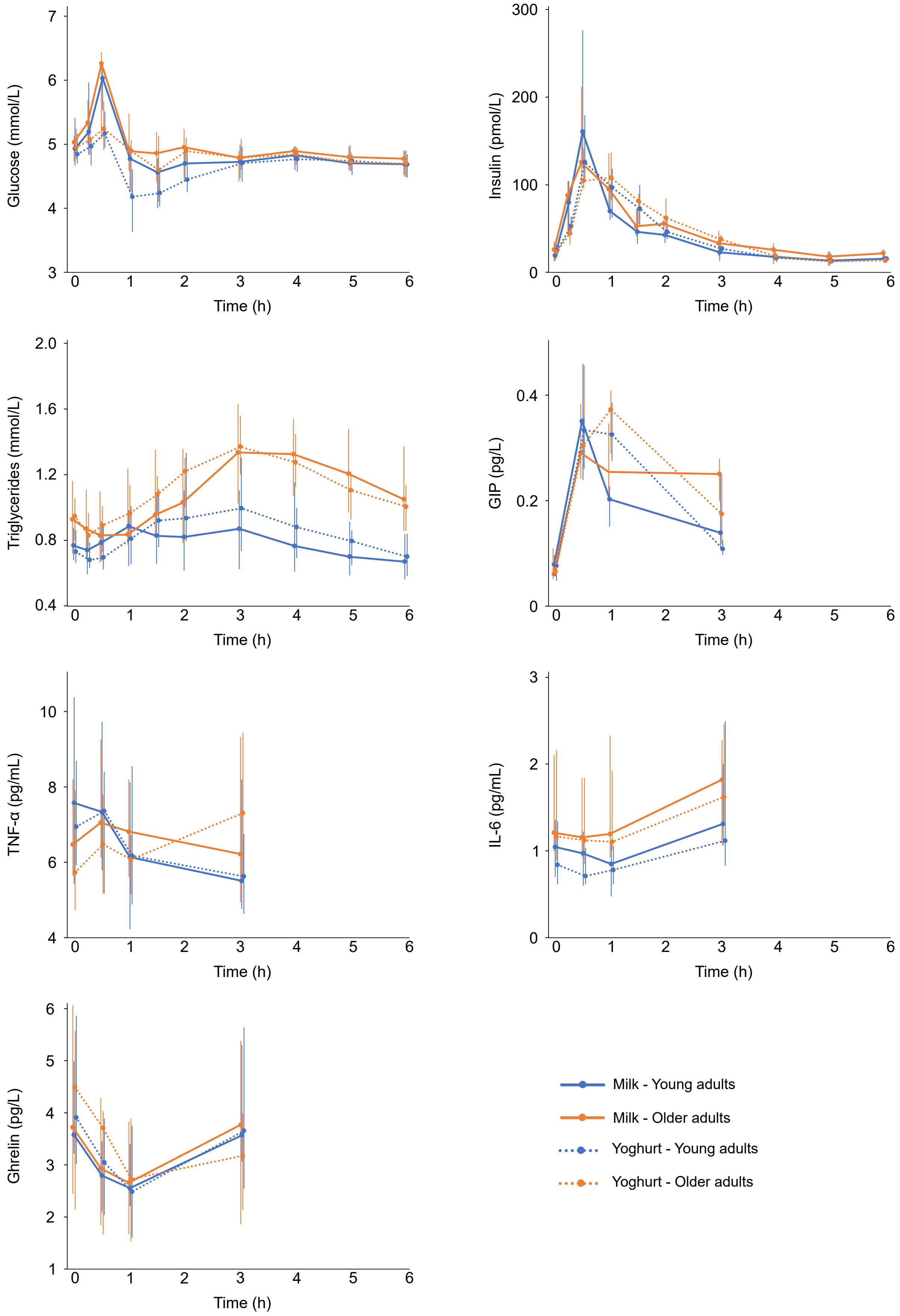

Figure S6. Absolute postprandial responses of biochemical markers to milk and yoghurt challenges. Line plots display postprandial responses as medians with interquartile ranges. GIP, glucose-dependent insulinotropic polypeptide; IL-6, interleukin-6; TNF-α, tumour necrosis factor-alpha.

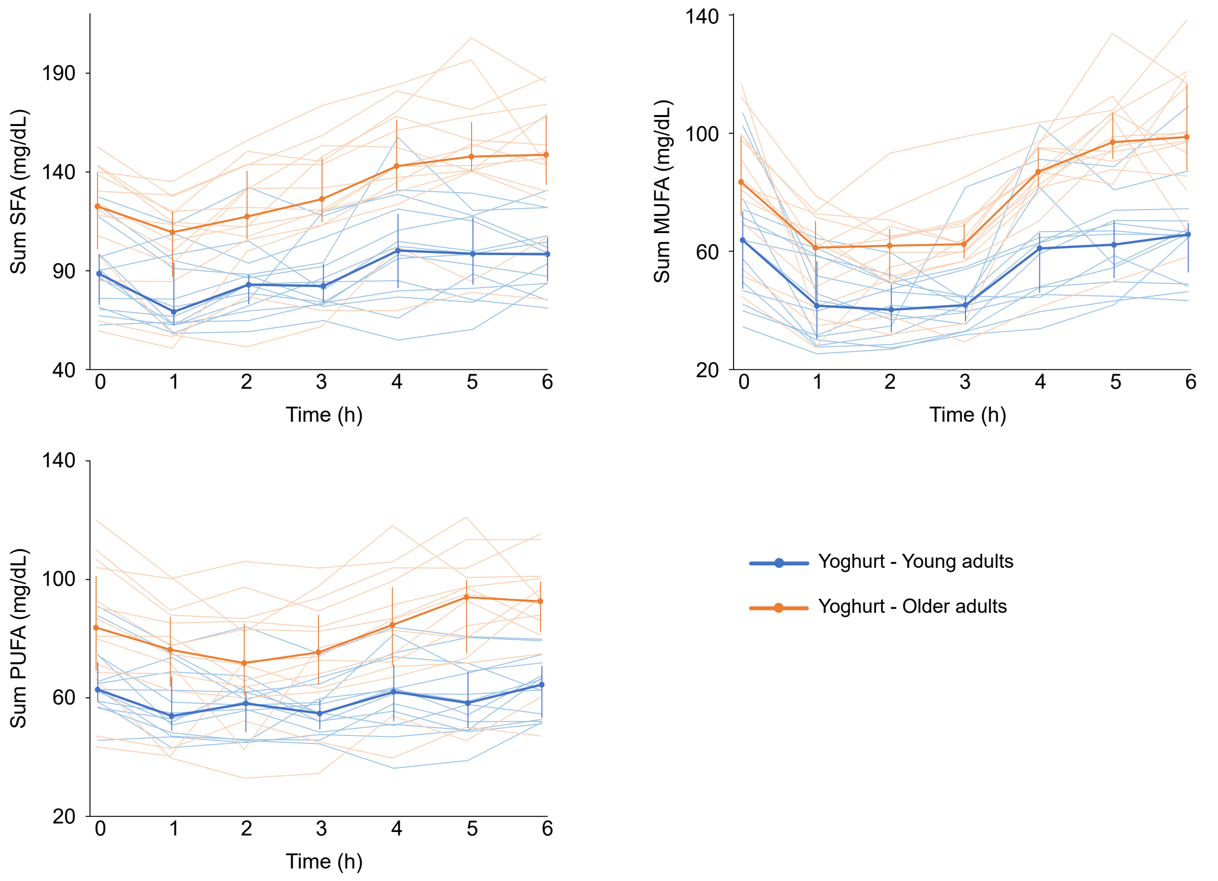

Figure S7. Absolute postprandial responses of FFA classes by age groups to yoghurt challenge.
Line plots display postprandial responses as medians with interquartile ranges. FFA, free fatty acid; MUFA, monounsaturated fatty acid, PUFA, polyunsaturated fatty acid; SFA, saturated fatty acid.
